# Enhancing utility and understanding of evidence based practice through undergraduate nurse education

**DOI:** 10.1186/s12912-017-0251-1

**Published:** 2017-09-29

**Authors:** Joanne Reid, Jordan Briggs, Susan Carlisle, David Scott, Claire Lewis

**Affiliations:** Queen’s University Belfast, Belfast, UK

## Abstract

**Background:**

The concept of evidence-based practice is globally relevant in current healthcare climates. However, students and teachers struggle with integrating evidence based practice effectively into a curriculum. This has implications for nurse education and in particular the way in which research is presented and delivered to students.

A new undergraduate Evidence Based Practice module (Evidence Based Nursing 1) was developed in a large University within the United Kingdom. It commenced in October 2014 running in year one of a 3 year undergraduate nursing programme. This study sought to formally evaluate attitudes and beliefs, knowledge level and utilization of evidence based practice though using two validated questionnaires: Evidence Based Practice Beliefs Scale© and Evidence Based Practice Implementation Scale©.

**Method:**

This was a pilot study using quantitative pre and post-test design. Anonymised data was collected from Year 1 undergraduate student nurses in the September 2014 intake (*n* = 311) at two time points. Time 1: pre-module in September 2014; and Time 2: post –module in August 2015. All data was collected via Survey Monkey.

**Results:**

Results demonstrate that the educational initiative positively impacted on both the beliefs and implementation of evidence based practice. Analysis highlighted statistically significant changes (*p* < 0.05) in both the Evidence Based Practice Beliefs Scale (7/16 categories) and the Evidence Based Practice Implementation Scale (13 / 18 categories).

**Conclusions:**

The significance of integrating evidence based practice into undergraduate nurse education curriculum cannot be underestimated if evidence based practice and its positive impact of patient care are to be appreciated in healthcare settings internationally.

## Background

Evidence based practice (EBP) is defined as (p.71) “‘the conscientious, explicit and judicious use of current best evidence in making decisions about the care of the individual patient. It means integrating individual clinical expertise with the best available external clinical evidence from systematic research’” [1]. Whilst this definition was developed in the context of evidence based medicine, it has come to define the general evidence based practice movement across all healthcare settings, including nursing. The full value and importance of EBP in nursing has been discussed previously [2] however there remains a reluctance by the profession to embrace EBP with many nurses reporting a lack of knowledge about what it constitutes and how to implement it.[3, 4]. This has led to an increased emphasis on EBP teaching within undergraduate nursing curricula, with mandates from professional regulatory bodies such as the United Kingdom’s (UK) Nursing and Midwifery Council (NMC) that it is taught throughout all 3 years of undergraduate nursing degree programmes within the UK.

Integrating evidence based practice when teaching research to undergraduate nursing students can help improve research knowledge.[5]. Nevertheless, evidence based practice remains three words with the potential to impart apprehension amongst many undergraduate student nurses.[6]. This current generation of undergraduate nursing students are among the first to have skills and knowledge in evidence based practice incorporated into their to education curriculum, combining teaching on research methods, research critique, application and evaluation in practice. Nurse educators therefore have potential to be change agents in the widespread future adoption of EBP by instilling knowledge and confidence in undergraduate nursing students about the importance and utilisation of evidence in their practice [7]. The direct link between evidence based practice and improved: quality of care; patient safety; and costs effectiveness underscores the importance of nurses being evidence based practitioners [8].

### Literature review to inform evidence based practice module development

Evidence based practice is not a new concept in nursing; indeed the Briggs report [9] suggested that nursing should become a ‘research based’ profession. Despite this, recent research undertaken with over 1000 newly qualified nurses across Europe suggested they are uncertain about their intention to use research evidence [10] and international literature suggests that students can still struggle to understand the relevance of evidence based practice to nursing [11]. Nonetheless, present-day nursing demands that nurses provide care that is based on best available evidence, as opposed to care based solely on tradition or authority [12]. To do so, nursing education providers need to ensure they build capacity in undergraduate nursing students to equip them with the skills to understand the importance and application of EBP.

Current research demonstrates that student nurses generally acknowledge the role that EBP plays in clinical practice. Indeed a recent study of undergraduate student nurses noted that most participants reported a positive attitude towards EBP, with a majority indicating that it was important for practice and quality of patient car [13]. Additionally, empirical evidence has demonstrated that undergraduate student nurses overwhelmingly support the notion that EBP can improve clinical practice.[14]. Despite this, undergraduate student nurses still favour EBP courses less than other core modules in their curriculum [14]. Additionally, there is a necessity for meticulous planning of how EBP is delivered in undergraduate curriculum. Indeed a recent study indicated that prior to a ‘reshaping of the perception of EBP’ course the majority of undergraduate student nurses had indicated that they did not fully grasp how to link EBP to clinical practice and that their general attitude had been negative towards EBP [15]. Thus when developing EBP educational initiatives, careful consideration must be given not only to EBP skills such as critiquing but also to the relevance of EBP to nursing.

### Developing a new undergraduate EBP module

The evidence above was instrumental for module coordinators (JR and CL) when designing a new EBP module (Evidence Based Nursing 1, EBN1) for year 1 of a 3 year undergraduate nursing degree programme. This was a core module, accounting for 40 CATS of an undergraduate year 1 programme, made up of 24 h of tutorials and 48 h of lectures. The coordinators took steps to ensure the content was grounded in practice. These steps included discrete slides per lecture on applicability to practice; a theory to practice link on each page of a specially designed EBN 1 e-resource; and a video or audio clip from a current member of staff discussing how their own research directly informs practice. This innovative approach and focus on practice ensured the module co-ordinators were utilising different skills to help teach EBP [14]. Indeed, various approaches to teaching such as large lectures, small group tutorials, an eResource, and the use of student response systems (a small key pad held by each student where they can select an answer to questions posed by the lecturer, answers from the entire class are displayed in bar charts immediate after the student selects their response) were integrated into the delivery of this module to provide teaching methods reflective of the subject area. The use of differing teaching strategies in EBP aligns with previous research which highlighted the need for multilevel support when delivering an EBP teaching program [16]. There is no doubt that EBP is a challenging subject for all those involved and that it presents significant challenges to academic institutions if it is to be delivered well. Many resources need to be in place for there to be a positive uptake by students and the planning of the module and development of resources was extremely time consuming during the first year of the module development. Additionally, as highlighted within the literature [17] the module coordinators were cognisant of the skill base of those teaching EBP to students in order to determine an awareness of complementary skills that will generally aid in the positive delivery of EBP. Thus within the EBN1 module the module coordinators were supported by a teaching team with expertise in both research methods and nursing practice.

## Aim

The overall aim of this pilot study was to use a pre-test post-testdesign to evaluate evidence-based practice at the start and on completion of year 1 undergraduate levelwithin a School of Nursing and Midwifery at a large University within the United Kingdom.

### Objectives

- To ascertain the attitudes and beliefs, knowledge level and utilization of evidence based practice of undergraduate students at the start of their degree programme and prior to undertaking evidence based practice training (Evidence Based Nursing 1 Module)
- To ascertain the attitudes and beliefs, knowledge level and utilization of evidence based practice of undergraduate students at the end of the first year of a degree programme which incorporated completing evidence based practice training (Evidence Based Nursing 1 Module)

### Framework

Evidence based practice is a seven step process [6]: (1) cultivate a spirit of inquiry; (2) formulate and answerable question; (3) Systematic search for research evidence; (4) appraisal of the validity, relevance, and applicability of the evidence; (5) integration of the research evidence with the clinical expertise of the practitioner and the wishes and desires of the patient / family; (6) Implementation of the evidence – based decision and evaluation of the outcomes; and (7) dissemination of the results. Numerous approaches have been proposed for the integration of EBP into undergraduate nurse education and the need for organisation support is also evidence [16]. Within this current study, the University sought to develop EBP modules across year 1, 2 and 3 of a 3 year degree in nursing program, this paper will evaluate year 1 which incorpatedEBN1.

## Methods

### Design

This pilot pre-test post-test study used a prospective descriptive exploratory approach using non-probability convenience sampling of all undergraduate student nurses taking the compulsory “Evidence Based Nursing Modules 1” at a large teaching university within the United Kingdom was conducted from September 2014 to September 2015. This research involved students anonymously completing two validated scales (Evidence Based Practice Beliefs Scale© (EBPB) and Evidence Based Practice Implementation Scale© (EBPI)) [18] online via SurveyMonkey. The EBPB scale contains 16 items that are scored on a five point Likert scale, ranging from strongly disagree (1) to strongly agree (5). The EBPI scale contains 18 items, and are scored on a 5 point frequency scale reflecting the amount of times students implemented each of the 18 items inside the last 8 weeks (0 = 0 times and 4 = > 8).

### Module

Evidence Based Nursing 1 is core module (1/4 of the year 1 undergraduate course) in undergraduate nursing education within the recruiting university. The module runs across the entirety of year 1 undergraduate nurse education. The module utilises a blended learning approach using lectures, small group teaching and an online eResource (with formative and summative assessment built in).

### Participants

The sampling frame for this study comprised all year 1 undergraduate student nurses in the September 2014 cohort (*n* = 311) undertaking the core module of “Evidence Based Nursing 1” at the recruiting University.

### Data collection

Data collection was at two time points: Time 1 prior to undertaking the Evidence Based Nursing 1 (September 2014); and Time 2 on completion of year 1 (August 2015) and Evidence Based Nursing Module 1). Students were emailed an invitation letter from the principle investigator (JR) with a link to the data collection site. All data was collected anonymously online via SurveyMonkey.

### Tools used

Demographic data including specialism studied (adult nursing, mental health nursing, or learning disability nursing), gender and age was collected from all participants. Students were also asked to complete two scales: (Evidence Based Practice Beliefs Scale© and Evidence Based Practice Implementation Scale© [18]. The reliability and validity of the scales have been demonstrated with both cronbach alpha and Spearmans-Brown reliability coefficients >.85 for each scale [18].

### Ethical considerations

School Research Ethics was granted in June 2014 to conduct this study. A clear description of the pilot pre-test post-test study was given both verbally and in writing to all potential participants. All potential participants were advised that: participation was voluntary; they had a right to withdraw from the project anytime; that their participation (or not) will not have an effect on their assessment or marks; only the investigator would have access to the raw data; that the results from this study would be published and presented at conferences; and that involvement would be anonymous. Potential participants were emailed a link to the SurveyMonkey site where all data was collected at Time 1 and 2. Submission of the completed questionnaire on Survey Monkey was taken as consent.

### Data analysis

All data was collected and automatically collated online via SurveyMonkey. Quantitative descriptive and non-parametric statistics (Mann-Whitney U test) were used to summarise and interpret the findings of this study. All data was collected anonymously via survey monkey and then transferred into SPSS for data analysis. All anonymised data was analysed independent of the chief investigator (JR), by a third party outside the research team (DS).

## Results

The pilot study has completed data collection and analysis of the first cohort of EBN1 students (September 2014 intake, *n* = 311) using validated tools [19]. Data was collected in September 2014 before commencing EBN1 (total number of responders = 124) and after completion of EBN1 (total number of responders =56) in August 2015. Table 1 display the demographic summaries of the participants within the study at Time 1 (before starting EBN1 module) and Time 2 (after completing EBN1 module) and highlight the majority of participants were female and studying adult nursing which is reflective of the student intake. Of note, the proportion of students from the mental health field was higher at the Time 2 data collection than Time 1. However, there was very little change in the overall numbers of mental health students who completed Time 1 date (*n* = 17) Time 2 date (*n* = 15).

**Table 1:** Specialism studied of participants

Topic studied	Time 1 (*n* = 124) N (%)	Time 2 (*n* = 56) N (%)
Adult nursing	75 (60%)	30 (53%)
Children’s nursing	22 (18%)	9 (16%)
Mental health nursing	17 (14%)	15 (27%)
Learning disability nursing	10 (8%)	2 (4%)

A Mann-Whitney U test was completed to determine if there was a difference between the Time 1 and Time 2 EBPI and EBPB results. Assumptions of the Mann-Whitney U Test [20] were met. For example: dependent variables were ordinal; groups were unmatched; distributions of Time 1 and Time 2 groups were similar, assessed by visual inspection via SPSS.

Analysis produced overall positive results as can be seen from Table 2 (Evidence Based Practice Beliefs Scale Results) and Table 3 (Evidence Based Practice Implementation Scale Results). Indeed, there were statistically significant results in 7 out of 16 categories and 13 out of 18 categories respectively. Statistically significant results are noted with an asterisk*.

**Table 2:** Evidence based practice beliefs scale results

Participants: Time 1 baseline n = 124; Time 2 Follow-up n = 56						
Question	Strongly disagree	Disagree	Neither agree nor disagree	Agree	Strongly agree	Sig.
1 I believe that EBP results in the best clinical care for patients	–	–	7 (8%)	43 (46%)	43 (46%)	P = .239
Time 2 - Follow-up	1 (2%)	1 (2%)	–	21 (38%)	29 (52%)	
2 I am clear about the steps of EBP	3 (3%)	22 (24%)	31 (34%)	33 (36%)	3(3%)	P = <.001^*^
Time 2 - Follow-up	1 (2%)	2 (4%)	6 (11%)	33 (59%)	10 (19%)	
3 I am sure that I can implement EBP	1 (1%)	5 (%)	27 (29%)	46 (50%)	13 (11%)	P = .104
Time 2 - Follow-up	1 (2%)	3 (6%)	6 (12%)	33 (64%)	9 (17%)	
4 Critical appraisal is an important step	1 (1%)	1 (1%)	15 (16%)	52 (56%)	24 (26%)	P = .005^*^
Time 2 - Follow-up	2 (%)	–	3 (6%)	21 (40%)	26 (50%)	
5 Evidence based guidelines can improve care	–	–	9 (10%)	43 (46%)	41 (44%)	P = .087
Time 2 - Follow-up	1 (2%)	–	1 (2%)	20 (39%)	30 (58%)	
6 I can search for evidence in a time efficient way	3 (3%)	–	32 (34%)	43 (46%)	15 (16%)	P = .594
Time 2 - Follow-up	–	7 (14%)	13 (25)	24 (46%)	8 (15%)	
7 I can overcome barriers in implementing EBP	1 (1%)	–	27 (29%)	58 (62%)	7 (8%)	P = .131
Time 2 - Follow-up	1 (2%)	6 (12%)	15 (29%)	25 (48%)	5 (10%)	
8. I can implement EBP in time efficient way	1 (1%)	1 (1%)	25 (27%)	57 (61%)	9 (10%)	P = .002^*^
Time 2 - Follow-up	2 (4%)	10 (19%)	14 (27%)	24 (46%)	2 (4%)	
9. EBP will improve the care I deliver	–	–	10 (11%)	46 (50%)	36 (39%)	P = .631
Time 2 - Follow-up	1 (2%)	1 (2%)	2 (4%)	26 (50%)	2 (4%)	
10. I am sure how to measure outcomes	3 (3%)	26 (28%)	41 (45%)	18 20%)	4 (4%)	P = .095
Time 2 - Follow-up	2 (4%)	13 (26%)	12 (24%)	22 (43%)	2 (4%)	
11. EBP takes too much time	11 (12%)	28 (31%)	52 (57%)	–	–	P = .037^*^
Time 2 - Follow-up	8 (15%)	28 (54%)	16 (31%)	–	–	
12. I can access best EBP resources	–	6 (7%)	41 (45%)	41 (45%)	4 (4%)	P = .012^*^
Time 2 - Follow-up	1 (2%)	3 (6%)	12 (23%)	32 (62%)	4 (8%)	
13. I believe EBP is difficult	4 (4%)	34 (37%)	54 (59%)	–	–	P = .764
Time 2 - Follow-up	10 (19%)	31 (60%)	11 (21%)	–	–	
14. I know how to implement and effect practice changes	–	29 (32%)	50 (54%)	12 (13)	1 (1%)	P = .007^*^
Time 2 - Follow-up	–	13 (25%)	18 (35%)	20 (39%)	1 (2%)	
15. I am confident I can implement where I work	1 (1%)	17 (19%)	37 (40%)	33 (36%)	4 (4%)	P = .764
Time 2 - Follow-up	2 (4%)	9 (17%)	17 (33%)	22 (42%)	2 (4%)	
16. I believe the care I deliver is evidence based	–	2 (2%)	44 (36%)	38 (41%)	8 (9%)	P = .007^*^
Time 2 - Follow-up	–	4 (8%)	7 (14%)	33 (65%)	7 (14%)

**Table 3:** Evidence based practice implementation scale results

Participants: Time 1 baseline n = 124; Time 2 Follow-up n = 56						
Question	0 times	<3 times	5 times	>5 < 8 times	>8 times	Sig
1.Use evidence to change my practice	46 (73%)	11 (18%)	3 (5%)	2 (3%)	1 (2%)	P < .001^*^
Time 2 - Follow-up	10 (22%)	24 (53%)	–		3 (7%)	
2.Critically appraised evidence	46 (73%)	12 (19%)	1 (2%)	2 (3%)	2 (3%)	P < .001^*^
Time 2 - Follow-up	14 (32%)	20 (46%)	–	6 (14%)	4 (10%)	
3.Generated PICO question	58 (92%)	4 (6%)	1 (2%)	–	–	P < .001^*^
Time 2 - Follow-up	16 (36%)	15 (33%)	–	12 (27%)	2 (4%)	
4.Informally discussed	46 (73%)	11 (17%)	4 (6%)	1 (2%)	1 (2%)	P < .001^*^
Time 2 - Follow-up	16 (36%)	15 (33)	–	10 (22%)	5 (9%)	
5.Collect data on patient problem	45 (71%)	11 (18%)	4 (6%)	2 (3%)	1 (2%)	P = .164
Time 2 - Follow-up	25 (56%)	17 (38%)	–	2 (4%)	1 (2%)	
6.Shared evidence with colleagues	50 (79%)	8 13%)	2 (3%)	2 (3%)	1 (2%)	P = .006^*^
Time 2 - Follow-up	24(53%)	15 (33%)	–	6 (13%)	–	
7.Evaluated outcomes of practice change	48 (77%)	10 (16%)	1 (2%)	3 (5%)	–	P = .151
Time 2 - Follow-up	29 (64%)	12 (27%)	–	4 (9%)	–	
8.Shared an EBP guideline with a colleague	50 (79%)	9 (14%)	1 (2%)	3 (5%)	–	P < .001^*^
Time 2 - Follow-up	18 (41%)	21 (48%)	–	2 (5%)	3 (7%)	
9.Shared evidence with patient, family	46 (73%)	12 (19%)	3 (5%)	2 (3%)	–	P < .001^*^
Time 2 - Follow-up	13 (29%)	23 (51%)	–	7 (16%)	2 (4%)	
10.Shared evidence with multi-disciplinary colleague	50 (81%)	10 (16%)	2 (3%)	–	–	P = .044^*^
Time 2 - Follow-up	29 (64%)	11 (24%)	–	4 (9%)	1 (2%)	
11.Read and critically appraised study	48 (77%)	10 (16%)	4 (6%)	–	–	P < .001^*^
Time 2 - Follow-up	17 (39%)	18 (41%)	–	7 (16%)	2 (5%)	
12.Accessed Cochrane	56 (90%)	3 (5%)	3 (5%)	–	–	P < .001^*^
Time 2 - Follow-up	12 (27%)	10 (22%)	–	17 (38%)	6 (13%)	
13.Accessed National Guidelines	55 (89%)	5 (8%)	2 (3%)	–	–	P < .001^*^
Time 2 - Follow-up	27 (60%)	12 (27%)	–	5 (11%)	1 (2%)	
14.Used EBP guideline	54 (86%)	7 (11%)	2 (3%)	–	–	P = .005^*^
Time 2 - Follow-up	28 (62%)	14 (31%)	–	3(7%)	–	
15.Evaluated care initiative	53 (84%)	6 (10%)	2 (3%)	–	2 (3%)	P = .294
Time 2 - Follow-up	33 (75%)	9 (21%)	–	2(5%)	–	
16.Shared outcome data	51 (81%)	5 (8%)	5 (8%)	2 (3%)	–	P = .432
Time 2 - Follow-up	33 (73%)	9 (20%)	–	3 (7%)	–	
17.Changed practice based on outcome data	49 (78%)	9 (14%)	3 (5%)	1 (2%)	1 (2%)	P = .169
Time 2 - Follow-up	29 (64%)	13 (29%)	–	3 (7%)	–	
18.Promoted use of EBP	50 (81%)	7 (11%)	3 (5%)	1 (2%)	1 (2%)	P < .001^*^
Time 2 - Follow-up	18 (40%)	18 (40%)	–	8 (18%)	1 (2%)	

Additionally, the results from this study can also be mapped onto the Learning outcomes for the EBN1 module, as displayed in Table 4 below. This is particularly useful for the module coordinators in planning future content delivery of EBN1.

**Table 4:** Mapping analysis onto EBN1 learning outcomes

Learning outcome from module proforma	Research Scale and Question which measured learning outcome	pre-post test analysis (Mann-Whitney Test)
Understand the meaning of evidence based practice	EBP Beliefs Scale, Question 2 *I am clear about the steps of EBP*	p = <.001
Develop an awareness of application to practice	EBP Implementation Scale, Question 14	
	*Used EBP guideline*	P = .005
	EBP Implementation Scale, Question 18	
	*Promoted the use of EBP*	p = <.001
Consider why professional nursing practice has to be evidence based	EBP Implementation Scale, Question 1	
	*Use evidence to change my practice*	p = <.001
	EBP Implementation Scale, Question 6	
	*Shared evidence with colleagues*	p = .006
Understand the concept of hierarchy of evidence	EBP Implementation Scale, Question 12	
	*Accessed Cochrane*	p = <.001
	EBP Implementation Scale, Question 13	
	*Accessed National Guideline*	p = <.001
Create a well-built clinical question using the PICO formula PICO – P = Patient / Problem, I = Intervention, C = Comparator, O = Outcome	EBP Implementation scale, Question 3 *Generated PICO Question*	P < .001
Gain a basic understanding of the nursing information environment, in order to lay a foundation for lifelong information management and searching skills.	EBP Beliefs Scale, Question 12	
	*I can access best EBP resource*	P = .012
	EBP Beliefs Scale, Question 14	
	*I know how to implement and effect practice changes*	P = .007
Understand basic data gathering and analysis methods	EBP Implementation scale, Question 11 *Read and critically appraised a study*	P < .001
Explain the decisions that influence the choice of methodological design	EBP Implementation scale, Question 2	
	*Critically appraised evidence*	P < .001
	EBP Beliefs Scale, Question 12	
	*Critical appraisal is an important step*	P = .005

## Discussion

This pilot pre-test post-test study sought to formally evaluate the attitudes and beliefs, knowledge level and utilization of evidence based practice in undergraduate students at the start of their degree programme and again at the end of their first year, which incorporated undertaking evidence based practice training (EBN1 Module)The students who participated in the research demonstrated that the after completion of their first year they had positively developed their understanding and utility of evidence based practice in nursing. Previous research in this field has shown that exposure to educational programmes does not significantly alter the students’ attitudes towards EBP [13]. This previously published study [13] evaluated a research education program and of the entirety of a year three undergraduate intake, only those that completed Time one data were invited to complete Time two. This current study evaluated the first year of an undergraduate programme which contained an EBP teaching initiative (EBN1) alongside three six-week clinical placements throughout year one. All year one undergraduate nursing students were invited to complete data collection at both time points. The current study demonstrates that the EBN1 educational initiative alongside clinical placements did alter both the beliefs (7/16 categories) and implementation (13/18 categories) of EBP for this cohort of undergraduate student nurses.

The absence of significant changes between Time 1 and Time 2 for the highly rated categories in the EBP beliefs scale such as: *“I believe that EBP results in the best clinical care for patients; Evidence based guidelines can improve care”;* may be due to a ceiling effect, with many of the student nurses reporting agreeability at Time 1. However there are many encouraging significant changes between Time 1 and Time 2 in the EBP implementation scale in categories such as: [I have] *“used evidence to change my practice”; “used a EBP guideline”;* indicating that even after 1 year of exposure to EBP education, alongside completing three six-week clinical placements, nursing students were able to use evidence and EBP guidelines to change their practice.

Significant changes in were also evident in items which questioned some of the more practical issues of implementing EBP such as critical appraisal (Question 2), generating a PICO question (Question 3), and accessing the Cochrane Database of Systematic Reviews (Question 12). This is promising given that a lack of skills in literature searching and critical appraisal is often cited as a barrier to effective implementation of EBP in nursing.[21, 22].

This pre-test post-test pilot study demonstrates that through completing year 1 of an undergraduate nursing degree which contains an educational module on EBP, undergraduate student nurses can increase their understanding and utility of EBP. However, this may not translate into their approach as subsequently qualified nurses in practice. This is indeed a notable issue as a recent study of over 1000 newly qualified nurses suggested that they have only a modest level of intention to utilise and integrate research evidence into their future practice [10]. Thus, future research needs to target qualified nurses (at different time points post qualifying) who have at undergraduate level completed EBP training at to ascertain how this educational initiative is transferred into post registration nursing care delivery.

### Limitations

As with any study there are limitations to this work. Firstly, this study did not include a control group and thus it is impossible to distinguish between the effect of providing the first year of a nursing degree programme without providing EBN1 and these results presented within this paper (first year of degree programme which incorporated EBN1). This data was anonymously submitted by students and therefore we can’t be sure that the people who provided data at follow-up actually gave baseline data; additionally responders may not be typical of the class as a whole. Additionally, the total numbers of undergraduate student nurses in this cohort was 311 and of this only 124 completed data collection at Time 1 and 56 at Time 2, thus data may not be comparable to all students. Finally, because we cannot look at individual pairs but have to look at pre and post groups the study may be under-powered, but we are constrained in this regard by the number of students per intake and the voluntary participation that is a requirement of this study. Nonetheless, these very positive set of findings, even given the limitations, illustrate that attending the EBN1 module has a major impact on EBP beliefs and implementation scores, which have direct applicability to the learning outcomes of the module.

## Conclusion

The drive towards equipping nurses with the skills of EBP is supported internationally by nursing registration bodies and global nursing associations, such as the International Council of Nurses who actively promote and support evidence-based practice. As nurse educators we have a unique opportunity to teach the nurses of the future the skills and necessity of being evidence based practitioners. Data from this study demonstrates that targeted education on EBP can enhance undergraduate student nurses understanding and utility of EBP. The EBN nursing modules that run within the recruiting University across years 1, 2 and 3 of the undergraduate program, focus on complementary and cumulative learning thus increasing student nurses capacity to work as evidence based practitioners. Further research is needed on how this education, delivered at undergraduate level, influences nursing care delivery post registration.

## Abbreviations

EBN1: Evidence based nursing 1
EBP: Evidence based practice
EBPB: Evidence based practice beliefs scale
EBPI: Evidence based practice implementation scale
NMC: Nursing and midwifery council
PICO: P = patient / problem, I = intervention, C = comparator, O = outcome
UK: United Kingdom
